# Dynamic Navigation in Dental Implantology

**DOI:** 10.15190/d.2023.17

**Published:** 2023-12-31

**Authors:** Lata Goyal, Hariram Sankar, Meghna Dewan, Yeshwanth Perambudhuru

**Affiliations:** ^1^Periodontics Division, Department of Dentistry, All India Institute of Medical Sciences, Bathinda, Punjab, India; ^2^Department of Dentistry, All India Institute of Medical Sciences, Bathinda, Punjab, India; ^3^Private Practice, Shimla, India; ^4^Former Scientist C, All India Institute of Medical Sciences, New Delhi, India

**Keywords:** Dynamic navigation, dental implants. Freehand approach, static navigation.

## Abstract

Implant placement for dental rehabilitation has gained more popularity among patients in the recent past. Dental Implants are the workhorse of dentistry. Previously, the implants were placed with the help of the traditional freehand approach. Even though the conventional technique was successful, it has his own shortcomings. Various methods have been introduced, like stent -guided implant placement and navigation guided implant placement, that enhance the precision of implant position. The three different methods for placing the implants are freehand approach, static navigation and dynamic navigation. Among these approaches, the dynamic navigation system is a promising technology in implant dentistry. The dynamic navigation system is being used successfully in various other fields and is well known for its accuracy. It gives an advantage to clinician by providing real-time three-dimensional position of implant and better clinical and patient related treatment outcomes. This review summarizes- the literature and evidence available on dynamic navigation, its potential application, advantages, disadvantages with future directions.

## SUMMARY

1. Introduction

2. Surgical Navigation - Registration

3. Different methods for placing implants

- 3.1 Freehand approach

- 3.2 Static Navigation

- 3.3 Dynamic Navigation

- 3.4 Freehand approach vs dynamic navigation surgery

- 3.5 Static guided surgery vs dynamic navigation surgery - Experienced vs non-experienced

4. Advantages of using dynamic navigation in implantology

5. Disadvantages of dynamic navigation

6. Importance of imaging technologies

7. Patient’s perspective

8. Future of implant dentistry

9. Conclusion

## 1. Introduction

Placing dental implants is one of the most popular dental procedures in recent years. The field of implantology is evolving day by day. The procedure is evolving gradually from the introduction of cone beam computed tomography (CBCT) imaging in treatment planning to the use of static implant guides in placing implants. From the introduction of Cone Beam Computed Tomography (CBCT) imaging in treatment planning to the use of static implant guides in placing implants, the procedure is evolving gradually. The procedure that utilizes static guides for implant positioning and drilling is known as the static navigation procedure. The next subsequent step in the field of implant dentistry is the introduction of dynamic navigation. Dynamic navigation technology permits the surgeon to work with the patient in real-time (i.e., the surgeon can gauge the orientation of the. implant drill in the bone using preoperative CBCT image on the screen)^^1^^. This fascinating technology has been successfully used in various medical fields, including neurosurgery, orthopaedics, surgical oncology, vascular surgery, otolaryngology and plastic surgery. In dentistry, it is used in various oral surgical procedures, such as midface fracture reduction, jaw resections, orthognathic surgery and treatment of temporomandibular joint problems^^2,3^^.

The purpose of dynamic navigation is not only the accurate placement of dental implants, but, more importantly, to ensure better clinical outcomes^^4,5^^. The better clinical outcome is achieved by placing implant in ideal position, effective and efficient prosthesis, aesthetics and efficient long-term peri-implant health. Its application in implantology is discussed in detail in this review article.

## 2. Surgical navigation

**Figure 1:**
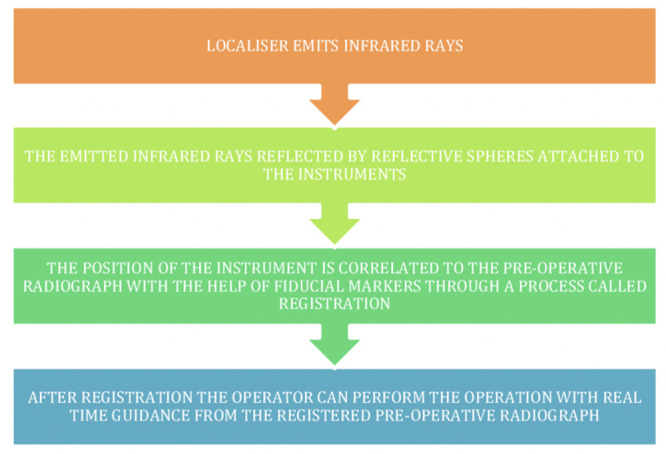
Figure 1. Navigation mechanism flowchart

**Figure 2:**
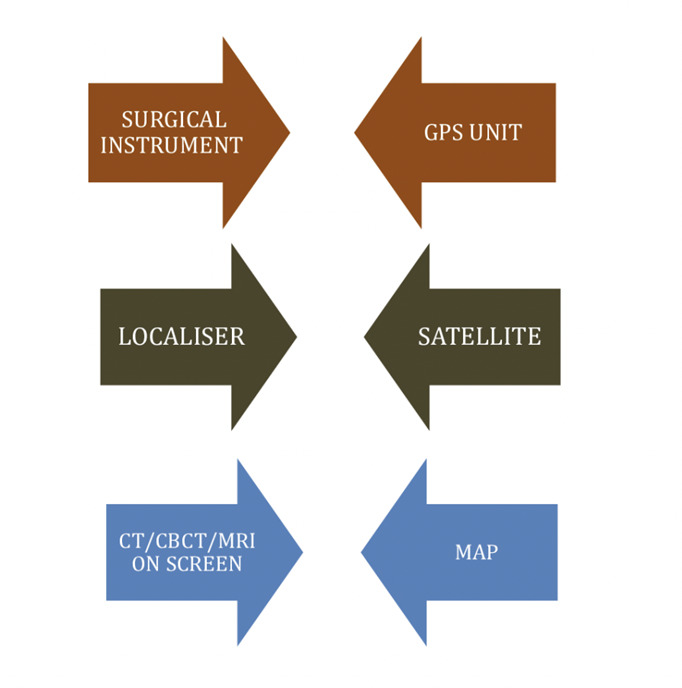
Figure 2. Navgation & GPS comparison

Surgical navigation system can be compared to a global positioning system (GPS). Like GPS, it consists of three basic components: a surgical instrument that can be compared to the GPS device, a satellite-like locator that controls GPS, and a Computed Tomography (CT) /Magnetic Resonance Imaging (MRI)/CBCT projected on the screen that is parallel to that of the map. GPS receives radio signals sent by satellites and combines these particulars with laden maps to determine the position. In surgical navigation, the locator (satellite) and the probe or surgical instrument (GPS unit) can related using mechanical, electromagnetic, ultrasonographic and optical means^^1^^. Most dynamic -navigation systems for implant surgery work with optical tracking. The optical tracking system can be either active or passive. The stereo camera traces the infrared light of the active tracking system. The reflective spheres in passive tracking systems reflect the infrared light from the source back to the camera. The most commonly used method is passive optical tracking. The light emitted by a source that is present above the patient. The light is reflected from tracking arrays above the patient and the instrument being tracked. The reflected light is picked up by stereo cameras above the patient. The preoperative image of the patient ’s paired with the patient position through a process called registration^^1,6^^ (Figure 1 and Figure 2).

*Registration*

Registration in surgical navigation is about establishing a relationship, linking the "real" coordinate system explained by the patient’s suggestion to the field and the "virtual" coordinate system of the image data. Registration is either point-based or uses surface matching routines. The surgeon virtually observes both the superimposed clinical situation and imaging data sets and can navigate both. During the registration process in implant surgery, even the depth of the drills must be registered. Registration is a process in which the preoperative CT scan is assigned to the patient using fiducial markers. The fiducial markers are stable anatomical indicators that can be cloned on the real and virtual patient. In implant surgery, the fiducial markers differ for dentulous and edentulous patients. In dentulous patients, the fiducial clip is firmly adapted to the patient's dentition. In this way, it helps to achieve a stable position and replicate the same position every time the patient brings their teeth in contact^^7,8^^. The thermoplastic material can be used as a reference clip after the patient's impression has been taken. For edentulous patients, the fiducials are placed in the patient's alveolar bone with small screws. The placement of the fiducial markers in edentulous patients is more invasive compared to dentulous patients^^1,6^^.

## 3. Different Methods for Placing Implants

### 3.1. Freehand Approach

The most commonly used technique is the freehand method. With this conventional method, the accuracy of the implants depends entirely on the skills and dexterity of the surgeon. The implant is placed by the surgeon using the opposing and adjacent teeth as a reference mark and some calibrated probes are used to measure whether appropriate height and width are present.

**Figure 3:**
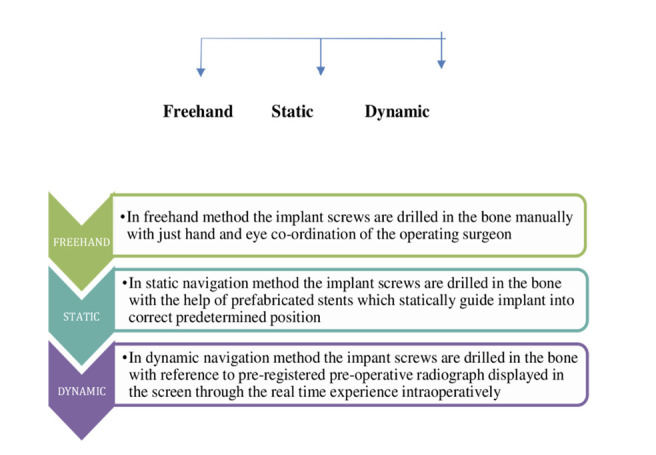
Figure 3. Different methods for placing implants

### 3.2. Static Navigation

The static guided approach uses various surgical templates for implant placement. Based on the material used, the stent can be either clear vaccuform stent which is easy to fabricate, but too flexible while placing implants, which further increases inaccuracy of implant position, chemical cure acrylic stent with lead strips, which is a diagnostic stent not used for surgery, self-cure acrylic with metal sleeves and disks, which is the most accurate but expensive, inflexible and self-cure acrylic with gutta percha filled channels, these are not as good as metal sleeves^^9,10^^. Based on support, surgical guides can be tooth supported, bone- supported or mucosa supported^^11^^. These surgical templates help maintain the angulation and position of the implants in the bone. There are plaster-based surgical templates that only maintain the position of the implants without taking into account the morphology of the bone^^12^^. There are also computerised templates that maintain the position of the implants, taking into account the bone morphology. The stents with metal tubes are designed and fabricated using CT -generated computer-aided design along a surgical system that uses coordinated instruments for placing stent- guided implants^^13^^.

### 3.3. Dynamic Navigation

Latest approach in placement of dental implants is dynamic navigated surgery. With this approach, implants can be placed dynamically or virtually in real time^^14,15^^. Based on the X-ray image projected on the monitor, the surgeon can see the exact position of the implant on. the monitor, so he/she can assign it in real time and navigate accordingly. This approach is definitely a better option, as the surgeon can track the depth, angulation and position of the implant throughout the procedure^^16^^. To determine whether dynamic navigation is really essential for successful implant surgery, freehand dynamic navigation and static navigation are compared. This helps dentists to make evidence-based decisions^^17,18^^.

### 3.4. Free-Hand Approach Vs Dynamic Navigation Surgery (Table 1)

The freehand method is still the most commonly used method for placing implants. It does not involve any form of 3-D guided treatment planning and carries more risk of inaccurate implant placement^^19^^. Inaccuracies are the main cause of various complications such as inferior alveolar nerve injury, adjacent root injury, membrane perforation-haematoma in the floor of the mouth, fracture of implant due to off-centre loading and increased prosthetic complexity^^20,21^^. Dynamic navigation can correct the inaccuracies and has been shown to be more accurate and better than the freehand approach in several studies. Implant accuracy has been measured using different units such as deviation in coronal, apical and angular directions^^22,23^^.

**Table 1:** Table 1. Freehand vs Dynamic navigation

S. N0	Aydemir CA, et al ^24^	March 2020	Type of study	Primary outcome	Secondary outcome	No of implants	Comparison	System used	Site operated	Conclusion
1	Aydemir CA, et al ^24^	March 2020	RCT	Accuracy	-	92	Dynamic navigation vsfreehand	NavidentClaroNa v technology,Toronto, Canada	Posterior maxilla	Navigation has better accuracythan freehand
2	Chen Z, et al ^25^	Dec 2018	Prospectiv e invitro	Accuracy	Experience	60	Freehand vs dynamic navigation	Navident ClaroNav technology,Toronto, Canada	Not specified	Dynamic navigation is a good implanttool
3	Kramer FJ, et al ^30^	Feb 2005	Prospectiv e invitro	accuracy	-	100	Freehand vs dynamic navigation	DenX, Israel	Maxillary anteriors	Navigation has superior outcome
4	Hoffmann J, et al ^31^	October 2005	Prospectiv e invitro	Accuracy	Experience	224	Dynamic navigation vsfreehand	Vector Vision, Brain lab,Germany	-	Dynamic > freehand
5.	Block MS, et al ^22^	Jan 2017	prospectiv e	Accuracy	-	100	Freehand vs static vsdynamic	X-guide X-Nav technology	Maxilla and mandible	Accuracy of static anddynamic same
6	Jorba-García A et al ^62^	Jan 2019	Prospectiv e in vitro	Accuracy	Experien ce of surgeon	36	Freehand vs dynamic navigation inexperience	Navident, ClaroNav technology,Toronto, Canada	Only mandible models	Successful method regardless ofexperience
7	Block MS, et al ^4^	July 2017	Prospectiv e study	Accuracy	-	714	Freehand vs dynamic navigation	Not mentioned	-	Accuracy of dynamic navigationbetter

In a randomised split-mouth control study conducted by Aydemir and Arisan in 32 patients, comparing freehand and dynamic, dynamic navigation device assistance provided an additional approximate accuracy of 0.7 mm linear and 5° angular^^24^^. In a cadaveric study of implant placement using flapless technique in the anterior maxilla conducted by Chen and Le et al, observed that the navigation method had greater accuracy than the freehand method^^25-27^^. Edelman et al, concluded that navigation technique may be more successful than the freehand method using a non-invasive method in a comparative study^^28,29^^.

A few laboratory studies conducted on plaster models also reflect the superiority of navigation over the freehand method in the matter of accuracy. Kramer et al. compared placement of maxillary single-tooth implants using the freehand method versus dynamic navigation, implant position variations were lower for implants placed with navigation (P < 0.05)^^30^^. In both the axial and transverse planes, implant angulations variations were lower for implants placed with a navigation protocol (P < 0.05). Difference in insertion depth of implants was smaller using navigation compared to conventional techniques. In an in vitro study, conducted by Hoffman et al., the accuracy of free hand method was inferior to the dynamic navigation^^31^^. Chang et al. observed the precision of dynamic navigation to be higher^^32,33^^. A randomised controlled trial conducted by Yotpibulwong et al in 2023, compared static and dynamic computer assisted implant surgery combined with all three surgical systems (freehand, guided and dynamic) in a total of 120 patients divided into four groups, the main parameter measured was. discrepancy in implant position and any other deviations measured at the level of platform, apex in all directions. It was found that combined static and dynamic surgery was more accurate when compared with freehand alone or static alone or dynamic alone^^34^^.

### 3.5. Static Guided Surgery Vs Dynamic Navigation Surgery (Table 2)

Static navigation, as the name suggests, uses static templates to guide the precise implant location and angulation. In other words, implant position cannot be changed intraoperatively with this method unless the stent is removed^^35^^. If the stent does not fit, the whole procedure has to be repeated. With this static guidance, the doctor can only use the same implant system^^20,29^^.

Static navigation, unlike the freehand method, uses computer-aided planning for implant placement, hence more accurate. Although static navigation is a potential replacement option for freehand surgery and has improved success rates, there are also some disadvantages^^36^^. There are some factors which influence implant surgery^^37,38^^. These include CBCT precision, the correspondence of the model to the CBCT file, the accuracy of the template fabrication, the tolerance of the template sleeve, the tissue support of the template, the precise fit of template, once fabricated modifications can’t be made on stent, maximun mouth, opening of the patient and surgeon’s experience with great learning curve in designing the guide^^29,39,40^^. Although dynamic navigation offers all these advantages, the accuracy of dynamic navigation compared to static navigation has not been proven statistically significant in most of the published studies. Implant site has crucial role in the success of both approaches^^41,42^^.

In a randomised control trial by Kaewsiri et al. comparing static and dynamic navigation, both showed comparable accuracy^^43,44^^. Yimraj et al. correlated static and dynamic systems with respect to accuracy, both techniques showed similar accuracy and parallelism between two implants^^45^^. Wu et al. observed static navigation to have comparable accuracy to dynamic navigation and also the experience did not have much influence on the static navigation technique^^46^^. Guzman et al. observed no significant statistical difference between the two techniques and came to the conclusion that both techniques are accurate^^27^^. Block et al. found the accuracy of static guidance comparable to that of dynamic navigation^^22,44^^.

*Experienced* *vs. Non-Experienced. *The accuracy of the implants will be higher with experienced surgeons. But with dynamic navigation surgery with the ability to work in real time, the new trainee surgeons have advantage and are able to place implants precisely^^47^^. Clinical experience has no significant impact on implant accuracy. Sun et al observed that with the help of dynamic navigation, the surgeons were able to place precise implants regardless of their clinical experience. This was also proven by several other in vitro studies^^48,49^^.

**Table 2:** Table 2. Static vs Dynamic Navigation

SN0	Authors	Year of publicati on	Type of study	Primary outcome	Secon dary outcome	No of implants	Comparison	System used	Site operated	Conclusion
1	Kaewsiri D, et al ^43^	May 2019	RCT	Accuracy	-	60	Static vs dynamic navigation	Straumann system	-	Dynamic =static
2	Yimarj P, et al ^45^	Dec 2020	RCT	Accuracy of position	paralle lism	60	Static vs dynamic navigation	IRIS-100; EPEDinc, Taiwan	Not specified	Similar accuracy between static anddynamic system
3	Wu D, et al ^46^	Dec 2020	Retros pectiv e study	Accuracy	Experience Implant site	38-dynamic 57- static	Static vs dynamic	DHC-DI3E,Suzhou digital healthcare, China	Teeth specified (anterior, premolar,molar)	Both accurate. No influence by experience and implant site
4	Mediavilla Guzmán A, et al ^27^	Dec 2019	RCT	Accuracy	-	40 (20x2)	Static vs dynamic	Navident, ClaroNav, Toronto, canada	Not specified	Both static and dynamic navigation allows accurate implant placement
5	Block M, et al ^22^	Jan 2017	prospe ctive	Accuracy	-	100	Freehand vs static vs dynamic	X-guide X-Nav technology	Maxilla and mandible	Accuracy of static and dynamic same

It was also highlighted that navigation can be used in training students for implants. Real-time correlation with the image on the screen allows students to get a better picture of the anatomy and also the angulation, position and depth of the hole during the learning phase^^50^^. Zhan et al. in his study attempted to evaluate the role of dynamic navigation in training dental students in implant placement^^51^^. This study concluded implant placement using dynamic navigation by students showed noteworthy improvement. They showed significant improvement in correcting implant deviations^^44,52^^. Pellegrino et al. observed that experienced surgeon’s had no influence on the accuracy by dynamic navigation. However, it was observed that the operating time was higher in the inexperienced surgeons compared to the experienced surgeons^^53^^ (Table 3).

## 4. Advantages of Using Dynamic Navigation in Implantology

Dynamic navigation in implantology has considerable advantages over both statically guided implants and the freehand method. Many recent clinical studies proved its benefits over other implant delivery methods^^54^^. Reliability of implant placement can be checked throughout the procedure, unlike freehand and static guided implants is single most benefit of this technique. The literature repeatedly points out the inaccuracies associated with this technique. In the case of a statically guided implant, if there is an error in the splint, the entire process is compromised. Another advantage of navigation is that most of the procedure is performed with the patient looking at the monitor. Even in the regions of aesthetic concerns like maxillary anterior by evaluating correct bucco-lingual, mesio-distal, apico-coronal dimensions of the bone and aesthetically and prosthetically planned implant can be placed using dynamic navigation system and favourable clinical and aesthetic outcomes can be expected^^55,56^^. In physiological rest position tongue is usually in rest against anterior part of hard palate, and this position has important role in speech and sleep apnea. Invasion of this space results in inadequate functional tongue space which will lead to tongue thrusting, open bite, rotations of teeth, trauma to lateral borders of tongue^^57-59^^. Risk of invading this space is possible with free handed surgery and poorly built static guided surgery which can be overcome by dynamic navigation system where there is possibility for intraoperative change of implant position^^60,61^^. Back pain, which is one of the most common occupational hazards of the operating dentist, can be avoided. Even in cases with restricted mouth opening, implants can be placed with minimal difficulty^^62,63^^. The patient's surgery can be scheduled and performed the same day without delay, without waiting for static splints to be made. With the advent of navigation, flapless surgery of implant placement can be advocated as the exact position of the drill in the bone is always visible^^64,65^^. Many studies have reported this method leads to sensible reduction of surgical time^^66-69^^.

**Table 3:** Table 3. Accuracy based on experience

A	B	C	D	E						
SN0	Authors	Year of publica tion	Type of study	Primary outcome	Secondary outcome	No of implants	comparison	System used	Site operated	Conclusion
1	Sun TM, et al ^48^	Dec 2019	Prospecti ve	Experienc e	Accuracy	30	Experienced vs inexperienced	AqNavi system, Taiwan and polarisVic ra optical trackingsystem	11,17,26,31,36,37region	1. Accuracy of navigation system not affected by experience2. Navigation system improves the operator accuracy
2	Pellegrino G, et al ^53^	Jan 2020	prospecti ve – in vitro	Accuracy	Operating time, Experience	112 (28x 4)	Accuracy in operators with varying levels ofexperience	ImplaNav,Bresmed ical, Sydney,Australia	Not specified	Reliable for both experienced and novice practioners
3	Sun TM, et al ^17^	Jan 2018	Prospecti ve (in vitro)	Accuracy	Learning curve acc tooperatio n site and operatingtime	150	Experienced vs inexperirnced	AqNavi system, Taiwan	Specified (6 sites)	1.The learning curve exhibited a learning plateau after 5 years. 2.Accuracy is same in maxilla andmandible
4	Stefanelli L, et al ^26^	Jan 2019	retrospec tive	accuracy	Impact of various factors on accuracy	231	First 50 implants vs last 50 implants	Navident, ClaroNav, Toronto, Canada	Not specified	1.Dynamic surgical navigation is accurate 2.Accuracy of dynamic navigation improves with experience in thetechnology
5	Golob Deeb J, et al ^52^	Nov 2019	RCT	Accuracy	Surgical time	70 (14x 5)	Accuracy of dynamic navigation guided implant among trainees	Navident dynamic guidance system	Both anterior and posterior (right and left)	Dynamic implant can improve implant surgical training in novice population

## 5. Disadvantages of Dynamic Navigation

The biggest disadvantage is the cost of the system and its accessories. Even for surgeons with good experience in implant placement, fully understanding the technique takes time and requires a learning curve. Another disadvantage is that edentulous patients require additional surgical exposure for fiducial placement^^70,71^^. One major complication which has been observed frequently using this technique is the loss of connection between the sensor and the camera^^72^^. The preference for dynamic over static navigation should therefore be justified.

## 6. Importance of Imaging Technologies

Both 2D and 3D imaging techniques have a crucial role in implant dentistry. Commonly used imaging technologies in implant dentistry are Radio-visiography (RVG), OPG, CBCT. Though intra oral radiographs and panoramic imaging considered to be suitable imaging techniques in dentistry, but they are not as accurate as CBCT which is 3D imaging technology. And also variations in magnification of panoramic imaging is seen in different OPG machines, so these are not completely reliable. According to International Commission on Radiological Protection low radiation exposure is noticed in intraoral and panoramic techniques when compared to CBCT which has greater exposure but less than CT. According to International Commission on Radiological Protection (ICRP) to minimization of this radiation exposure is done by following two ways i.e. justification and optimization, justification means radiographs should be advised only if necessary and not be used as a routine investigation. It basically means if benefits exceeds the risk with radiation only then radiograph should be advised after taking proper history and clinical examination^^73^^. Optimization means once decision of taking radiographs has been confirmed it should be as low as reasonably achievable. It is unimaginable to perform implant placement without radiographs, the reason being there is need for information about bone quality which is measured by using Hounsfield units, bone quantity in all dimensions and to measure distance from osteotomy site to nearest anatomical structures like inferior alveolar nerve canal, mental foramen, adjacent tooth structures, incisive canal and other pathologies if exists in mandible and nasal floor, nasal cavity, maxillary sinus with its floor, septa^^16^^. Advanced implant placing techniques like static and dynamic navigation systems are dependent on CBCT, in static navigation system with the available data from CBCT is used to 3D print the template which will provide depth, position and angulation of implant are constructed^^74,75^^. In dynamic navigation system template with implant reference markers are worn by the patient through-out the image acquisition and intraoperatively these reference markers provide constant information for the accurate precise placement of implants. These freehand technique, cause accurate implant placement is most important step for survival of implant in long-term^^76,77^^.

## 7. Patient’s Perspective

Common complications encountered in any dental surgery are pain and discomfort of the patient in between and after the surgery, swelling or edema post operatively, hypersensitivity, high patient’s expectations. Since everything is pre-planned and organised time taken in the dynamic navigation is less comparable to static guided surgery. In most recent studies no significant difference was found when it comes to post operative pain, swelling or edema which last not more than 2 weeks in all three surgical techniques and almost comparable patient satisfaction however slight discomfort was noticed in static guided surgery group while speaking^^36,78^^.

## 8. Future of Implant Dentistry

Dynamic navigation system is utilizing CBCT or other radiographic imaging to position implants, but chances of errors can’t be ruled out while using radio-diagnostic technology, Positioning errors are possible, even error in the device which marks the location is also a possibility^^79-81^^. High accuracy of robotic implant placement in replacing single tooth was shown recently in a case series by Yang et al^^82^^ where robotic implant surgery was performed to replace single missing tooth in 10 selected patients without any post operative complications or adverse surgical events, the study was success in establishing the accuracy of robotic implant surgery as an alternative method to novel dynamic navigation system. Bolding et al^^83^^ made an effort to demonstrate accuracy of haptic robotic guidance in placing implants. in completely edentulous arches for implant supported prosthesis, when compared with non- robotic methods, this robotic guidance has proven to be accurate in safely and effectively placing implants, and even in highly resorbed ridges in posterior maxilla which is considered to be complex situation robotic system has been used in a preliminary research by Li et al., where zygomatic implant are placed with minimal deviation and adequate accuracy without any deviation into lateral wall of maxillary sinus^^84,85^^. A new age technology, i.e. robotic technology for placing implants, has been under study to overcome all previous downsides for instance a phantom model study conducted by Chen J et al revealed angular deviation observed in robotic system was superior to dynamic navigation system and robotic technology has promising role in future dental implantology but at present it needs more clinical trials^^86^^.

## 9. Conclusion

Dynamic navigation in implant surgery is undoubtedly more accurate and has success rates. The superiority of navigation over the free-hands approach is significantly higher. The static guided/static navigation approach in implant surgery is more accurate compared to the freehand approach. There are several factors that affect the reliability of static navigation. The accuracy of both dynamic and static navigation is statistically comparable in various studies. The cost of the dynamic navigation system and accessories is comparatively expensive. Limited evidence has been seen to determine better aesthetics with dynamic navigation. Placement of dental implants with conventional or dynamic navigation protocols resulted in similar postoperative levels of patient satisfaction, oedema and pain medication. So, in the future, we need more studies with a large sample size to justify the use of dynamic navigation in clinical practice for placing regular dental implants.
